# Severe Human Granulocytic Anaplasmosis Transmitted by Blood Transfusion

**DOI:** 10.3201/eid1808.120180

**Published:** 2012-08

**Authors:** Matjaz Jereb, Blaz Pecaver, Janez Tomazic, Igor Muzlovic, Tatjana Avsic-Zupanc, Tanja Premru-Srsen, Snezna Levicnik-Stezinar, Primoz Karner, Franc Strle

**Affiliations:** University Medical Center Ljubljana, Ljubljana, Slovenia (M. Jereb, B. Pecaver, J. Tomazic, I. Muzlovic, T. Premru-Srsen);; Institute of Microbiology and Immunology, Ljubljana (T. Avsic-Zupanc);; and Blood Transfusion Center of Slovenia, Ljubljana (S. Levicnik-Stezinar)

**Keywords:** human granulocytic anaplasmosis, severe form, blood transfusion, zoonosis, bacteria, *Suggested citation for this article*: Jereb M, Pecaver B, Tomazic J, Muzlovic I, Avsic-Zupanc T, Premru-Srsen T, et al. Severe human granulocytic anaplasmosis transmitted by blood transfusion. Emerg Infect Dis [serial on the Internet]. 2012 Aug [*date cited*]. http://dx.doi.org/10.3201/eid1808.120180

## Abstract

A 36-year-old woman acquired severe human granulocytic anaplasmosis after blood transfusion following a cesarean section. Although intensive treatment with mechanical ventilation was needed, the patient had an excellent recovery. Disease caused by *Anaplasma phagocytophilum* infection was confirmed in 1 blood donor and in the transfusion recipient.

Human granulocytic anaplasmosis (HGA), an emerging tickborne zoonosis caused by *Anaplasma phagocytophilum*, has been recognized in the United States since 1994 and in Europe since 1996 (*1*,*2*). Most patients acquire *A. phagocytophilum* infection by tick bite, although individual cases of nosocomial, perinatal, and transfusion-associated transmission have been reported (*3*–*5*). We report a case of severe HGA acquired from blood transfusion.

## The Case-Patient

On August 26, 2010, a 36-year-old woman, 29 weeks pregnant without underlying chronic illness, was admitted to the University Medical Center Ljubljana with preeclampsia and restriction of intrauterine growth. Because her previous pregnancy ended in spontaneous abortion, the patient was monitored closely in an inpatient setting. On September 15, an elective cesarean section was performed. Later that day, hemorrhagic shock developed. Surgical revision of the source of the blooding was performed, and she received 6 units of packed erythrocytes and 2 units of fresh frozen plasma, originating from 6 donors. Ten days later, on September 25, the patient became febrile, which was associated with an elevated C-reactive protein level and mild abnormalities in liver enzyme levels, but with no signs of localized infection (Table 1). Antimicrobial drug therapy with amoxicillin/clavulanic acid was initiated, but the regimen was changed after 3 days to gentamicin and metronidazole because the high fever did not abate. At that time, a chest radiograph revealed mild interstitial edema, and a vaginal ultrasound showed no abnormalities.

**Table 1:** Blood test results for a patient with severe human granulocytic anaplasmosis, Slovenia, 2010*

Date, 2010	CRP, mg/L	PCT, µg/L	Leukocytes, 10^9^ cells/L	Band cells, %	Erc, 10^12^ cells/L	Hb, g/L	Pt, 10^9^/L	LDH, μkat/L	AF, μkat/L	AST, μkat/L	ALT, μkat/L	GGT, μkat/L
Sep												
13	<3	ND	8.2	ND	4.23	113	230	2.67	1.8	0.46	0.53	0.19
16	45	ND	11.1	ND	4.57	127	142	3.74	1.5	0.78	0.53	0.23
25	97	ND	9.2	ND	4.76	128	258	ND	2.54	1.39	1.65	ND
27	95	0.75	6.2	ND	3.91	106	80	6.1	3.56	1.39	1.12	2.67
28	120	0.73	9.1	48	3.67	104	39	8.28	5.3	1.9	1.17	3.07
29	167	0.98	10	23	3.82	106	21	9.23	6.11	2.74	1.33	2.98
30	121	1.02	9.4	15	3.79	106	11	13.6	5.75	3.69	1.36	2.95
Oct												
1	88	0.83	6	10	3.92	111	21	18.12	5.07	4.06	1.29	3.28
2	60	1	12	10	3.94	104	50	23.2	4.3	4.86	1.48	3.8
5	34	0.23	13.9	3	3.2	91	52	16.03	3.33	1.8	1.4	4.06
6	15	0.19	14.3	2	3.38	97	141	10.48	2.72	1.2	1.52	3.21
7	4	ND	12	2	3.52	101	210	7.41	2.49	0.97	1.99	3.11
8	ND	ND	11.5	0	3.54	104	275	6.03	2.23	0.73	1.83	2.77
10	<3	ND	8.5	0	3.96	107	401	5.65	2.11	0.62	1.5	2.62

*CRP, C-reactive protein (reference <5 mg/L); PCT, procalcitonin (reference <0.5 µg/L); leukocytes, leukocyte count (reference 4–10 × 10^9^ cells/L); Erc, erythrocyte count (reference 4.2–5.4 x 10^12^ cells/L); Hb, hemoglobin (reference120–160 g/L); Pt, platelets (reference 140–340 × 10^9^/L); LDH, lactate dehydrogenase (reference <4.12 μkat/L); AF, alkaline phosphatase (referrence <1.74 μkat/L); AST, aspartate transaminase (reference <0.52 μkat/L); ALT, alanine transaminase (reference <0.56 μkat/L); GGT, γ-glutamyl transferase (reference <0.63 μkat/L); ND, not determined.

The patient's condition deteriorated further, and on September 27 she was transferred to an intensive care unit. Tachypnea (30–40 breaths/min) without hypoxia, tachycardia (120 beats/min), elevated temperature (37.8°C), and hypotension (90/60 mm Hg) were recorded at admission. Antimicrobial drug therapy was changed to imipenem, azithromycin, and vancomycin. Computed tomography scan of the chest showed consolidation in the lower right lobe. Blood cultures and other relevant microbiological tests remained negative for infectious agents. Antiphospholipid syndrome was suspected, and treatment with corticosteroids, immunoglobulins, and heparin was initiated. However, corresponding tests did not confirm the diagnosis. Drug therapy was changed to piperacillin/tazobactam, daptomycin, and azithromycin.

The fever continued, laboratory test results worsened (Table 1), and acute respiratory distress syndrome (ARDS) developed. Bone marrow examination, performed because of persistent thrombocytopenia, showed reactive changes. Because of the febrile illness associated with laboratory indicators of inflammation, presence of thrombocytopenia, and elevation of transaminases, as well as the ineffectiveness of treatment, a working diagnosis of HGA was posed, and doxycycline was added to the treatment regimen on October 1.

The diagnosis was confirmed by demonstration of morulae on examination of whole blood smears by microscopy (Figure), by a positive PCR for DNA coding 16S rRNA of *A. phagocytophilum* in whole blood, and later by seroconversion to *Anaplasma* antigens (Table 2). Morulae and *A. phagocytophilum* DNA were also detected in bone marrow biopsy samples (*6*,*7*). In addition, all samples positive by PCR were tested for the *groESL* operon of *A. phagocytophilum,* and reliability of products was confirmed by direct sequencing. On the second day of doxycycline treatment, respiratory distress progressed further and artificial ventilation was necessary. However, the next day the patient experienced dramatic improvement; on the fourth day after initiation of doxycycline, the breathing tube was removed, and her later clinical course was uneventful. She was discharged at the end of a 14-day treatment course of doxycycline, and at follow-up visits she reported no difficulties.

**Figure:**
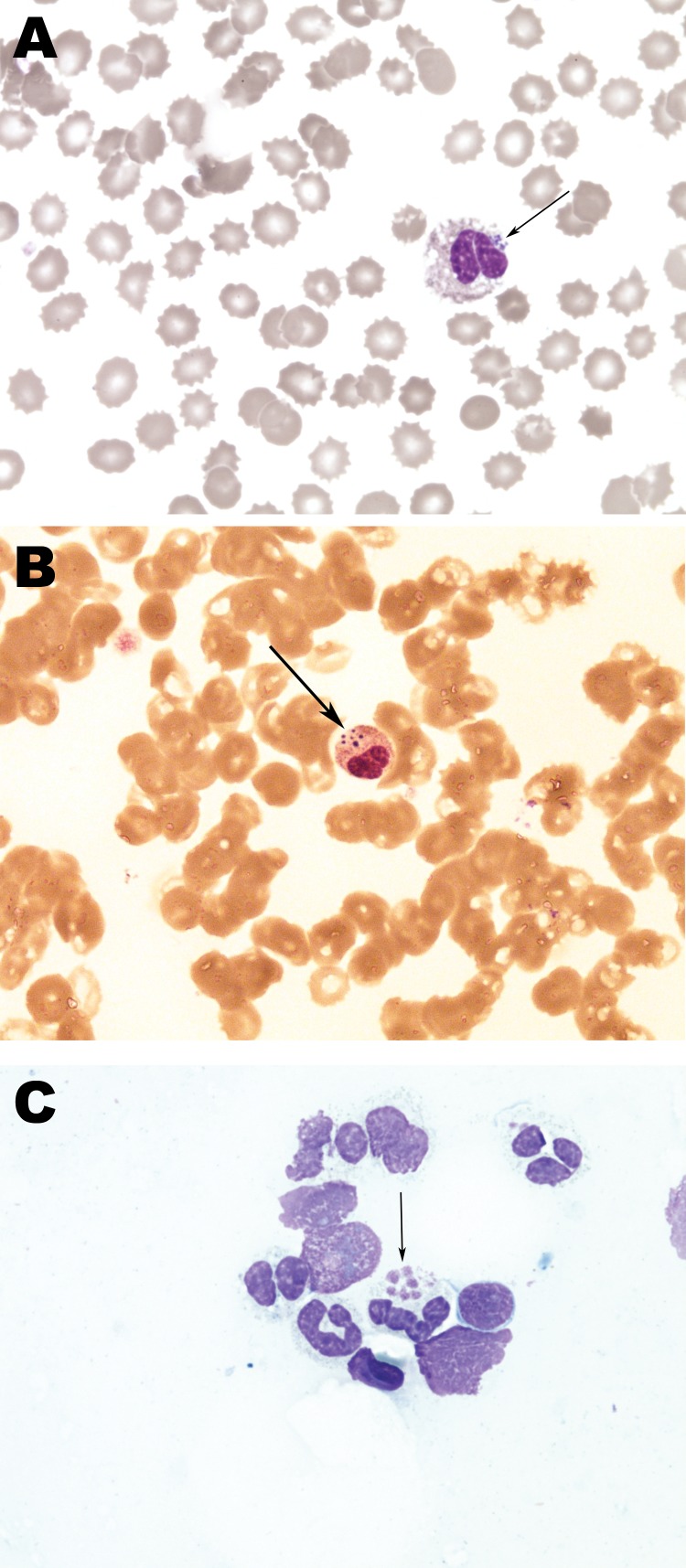
Histopathology slides from 36-year-old woman with human granulocytic anaplasmosis, Slovenia, 2010. Peripheral blood smear (A, B); bone marrow smear (C). Modified Giemsa staining, original magnification ×1,000. Morulae (clusters of *Anaplasma phagocytophilum* in granulocytic leukocytes) are indicated by arrows. In Europe, morulae have been reported in only 1 patient (*6*), but they are a relatively common observation in the United States, associated predominately with severe cases of human granulocytic anaplasmosis (*7**).*

**Table 2:** Results of PCR and IFA for IgG against *Anaplasma. phagocytophilum* for index patient and blood donor, Slovenia, 2010*

Date, 2010	Patient				Donor		
	PCR	IFA	Remarks		PCR	IFA	Remarks
May 12†					–	–	118 d before index donation
Aug 28–29					ND	ND	Febrile illness
Sep							
7†					+	>1,024	Index donation
15	–	–	Transfusion				
25	ND	ND	Onset of febrile illness				
Oct							
1	+‡	–	Treatment with doxycycline Oct 1–14				
4	+	128					
6	+	512					
9	+	ND					
10	–	ND					
12	–	512					
19	ND	512					
20					–	>1,024	43 d after index donation
Nov 23	ND	>1,024	No reports of problems				

*Results for IFA are shown in titers. IFA, immunofluorescence assay; ND, not done; –, negative; +, positive. †Retrospective testing of stored blood specimen. ‡DNA detected in stored bone marrow–biopsy specimen.

Because the patient denied having been bitten by ticks, had not left her house for several weeks before admission to the hospital on August 26 because of a complicated pregnancy, was continuously hospitalized for 30 days before the onset of fever on September 25, and received transfusions during her hospital stay, transfusion-associated transmission of HGA was suspected and searched for. Blood taken from the patient for pretransfusion cross-matching on September 15 tested negative by PCR and by immunofluorescence assay for antibodies against *A. phagocytophilum*. Stored plasma samples from all 6 blood donors, frozen on the day of donation (2 donated blood on August 10, 4 on September 7, 2010), were tested for antibodies against *A. phagocytophilum* and the presence of corresponding DNA. The results were negative for all but 1 donor. This 42-year-old man, a regular blood donor who lived in a region where sporadic HGA cases had been established (*8*), reported being an outdoor person who received several tick bites every year (the most recent in July 2010). He donated blood twice in 2010, on May 12 and September 7; blood obtained at the latter visit was transfused as packed erythrocytes to the patient reported here. At the end of August, a self-limited illness had developed in the donor with fever (39°C), myalgia, and arthralgia (Table 2).

## Conclusions

HGA is an acute febrile illness that causes headache, myalgia, malaise, elevated levels of C-reactive protein and serum transaminases, leukopenia, and thrombocytopenia; the disease seems to have milder manifestations in Europe than in the United States (*8*,*9*). The fatality rate is <1% (*9*), although a literature search did not reveal any report of a fatal case in Europe. The patient fulfilled the criteria for proven HGA (*10*). She had an acute febrile illness with thrombocytopenia, *A. phagocytophilum* infection demonstrated by the presence of corresponding DNA in plasma and bone marrow in conjunction with seroconversion, and spectacular improvement after treatment with doxycycline was instituted. The course of her illness was severe and encompassed pneumonia, ARDS, and the need for treatment in the intensive care unit, including mechanical ventilation. Although cough has been reported in 19% of patients with confirmed HGA cases in the United States, pneumonia or ARDS has been documented in only 1% (9). In Europe, pneumonia was recorded for just a few cases, and no data on respiratory failure exist (*11*).

In our patient, pregnancy, cesarean section, blood loss after the operation, an additional surgical procedure, corticosteroid treatment, and an interval of 6 days before correct diagnosis and treatment could have contributed to the severity of her illness. It is also possible that infection acquired through transfusion results in a more severe illness than infection after the bite of an infected tick. However, only a few reports of presumed transmission of *A. phagocytophilum* from sources other than ticks have been published (*3*–*5*,*12*). In a previous single report of transmission by blood transfusion (*5*), the evidence that HGA was acquired through the transfusion was convincing, but the report could not prove that the patient was free of *A. phagocytophilum* infection beforehand.

For the patient reported here, findings exclude tick transmission and convincingly favor transfusion-associated transmission of *A. phagocytophilum*. The latter was confirmed by the presence of *A. phagocytophilum* DNA in stored plasma specimens of 1 of the 6 blood donors. This donor, who had negative test results for the bacteria 4 months earlier, reported having had an acute self-limited febrile illness 2–3 weeks before blood donation. This infection probably resulted in severe acute HGA in the patient reported here.

*A. phagocytophilum* remains viable under refrigeration conditions at 4°C for up to 18 days, enabling potential transmission of infection by blood transfusion (*13*). This case of transfusion-associated HGA in Europe is practical evidence of such transmission and corroborates findings from the United States (*5*) that transfusion-associated febrile illness with thrombocytopenia could be caused by *Anaplasma* infection. Because transfusion-associated HGA appears to be very rare, routine screening of blood donors for the presence of *A. phagocytophilum* genome is not likely to be cost-effective. Nevertheless, when febrile illness associated with leukopenia or thrombocytopenia develops in a patient after transfusion, testing for infection with *A. phagocytophilum* may be beneficial.
